# ClpP Protease, a Promising Antimicrobial Target

**DOI:** 10.3390/ijms20092232

**Published:** 2019-05-07

**Authors:** Carlos Moreno-Cinos, Kenneth Goossens, Irene G. Salado, Pieter Van Der Veken, Hans De Winter, Koen Augustyns

**Affiliations:** Laboratory of Medicinal Chemistry, University of Antwerp, Universiteitsplein 1, B-2610 Antwerp, Belgium; carlos.morenocinos@uantwerpen.be (C.M.-C.); kenneth.goossens@uantwerpen.be (K.G.); irenegarciasalado@gmail.com (I.G.S.); pieter.vanderveken@uantwerpen.be (P.V.D.V.); hans.dewinter@uantwerpen.be (H.D.W.)

**Keywords:** antibiotics, antivirulence, ClpP

## Abstract

The caseinolytic protease proteolytic subunit (ClpP) is a serine protease playing an important role in proteostasis of eukaryotic organelles and prokaryotic cells. Alteration of ClpP function has been proved to affect the virulence and infectivity of a number of pathogens. Increased bacterial resistance to antibiotics has become a global problem and new classes of antibiotics with novel mechanisms of action are needed. In this regard, ClpP has emerged as an attractive and potentially viable option to tackle pathogen fitness without suffering cross-resistance to established antibiotic classes and, when not an essential target, without causing an evolutionary selection pressure. This opens a greater window of opportunity for the host immune system to clear the infection by itself or by co-administration with commonly prescribed antibiotics. A comprehensive overview of the function, regulation and structure of ClpP across the different organisms is given. Discussion about mechanism of action of this protease in bacterial pathogenesis and human diseases are outlined, focusing on the compounds developed in order to target the activation or inhibition of ClpP.

## 1. ClpP Function and Regulation

In every domain of life, cells and organelles are full of proteins, many of which are intrinsically disordered or in the process of folding [1]. Thus, proteolysis is an essential cellular activity, mediating protein turnover and the degradation of undesired or defective proteins from the intracellular environment. Since peptide bonds in an unstructured polypeptide are fairly sensitive to proteolysis, indiscriminate proteases are not present in the cytoplasm of archaea, bacteria and eukaryotic organelles. Instead, selected proteins in these intracellular environments are arrested, unfolded and degraded by proteolytic machines. These specific enzymes belong to the ATPases associated with diverse cellular activities (AAA+) superfamily, due to the presence of an AAA+ unfoldase partner in charge of the recognition of the desired substrates and the ATP-dependent mechanical unfolding of the targeted protein before its translocation to the proteolytic active site [2,3].

These proteolytic machines, enable the cell to keep proteostasis and to alter the protein levels to adapt to changing environments. The 26S proteasome present in eukaryotic cells is possibly the best known of these cellular machines [4]. AAA+ proteases present both in bacteria and eukaryotic organelles include FtsH, Lon, HsIUV, ClpPXP, ClpAP and ClpCP [2]. A protease found in all three domains of life is 20S peptidase, which associates with different AAA+ unfoldases such as Rpt1-Rpt6 ring of the 26S proteasome, proteasome-activating nucleotidase (Pan) and *Mycobacterium* proteasome-associated ATPase (Mpa) [4,5]. In bacteria, Lon and ClpP are estimated to carry out around the 80% of cellular proteolysis [6,7]. Coexistence of a number of AAA+ proteases is present in every species. Importance of their role and overlap of their functions determine the essentiality of each of them in every given organism.

ClpP is well characterized in many species, and it is always involved in the proteolysis of defective and misfolded proteins. More than 60 substrates of *Escherichia coli* ClpP were identified when using an inactive mutant as a trap [8]. These targeted proteins were involved in a variety of processes including transcription regulation, metabolism, damage repair and cell division. A sodium dodecyl sulfate polyacrylamide gel electrophoresis (SDS-PAGE) showed a similar selection in *Bacillus subtilis*, comparing wild-type (WT) and *clpP*-defective mutant (Δ*clpP*) strains [9]. In these and other bacteria, such as *Staphylococcus aureus*, [10] ClpP function affects greatly the degradation of proteins implicated in stationary phase adaptation, nutrient starvation, heat-stress response, biofilm formation, cell motility, cell-cycle progression or metabolism [11,12].

Although the targeted proteins are not the same in each of the organisms, the substrate identification in model bacteria shows the relevance of its function when regulating bacterial proteome. The important role that ClpP plays in the bacterial infectivity and virulence is explained by the range of processes in which its substrates are involved, pointing at ClpP as a prime target for antivirulence drug development [13,14,15,16,17].

This protease is also present in human mitochondria, where it is crucial for protein homeostasis and also plays a relevant role in the degradation and regulation of enzymes related with cellular metabolic pathways such as the electron transport chain [18,19,20]. ClpP overexpression has been associated with a broad range of carcinomas during the last years [21,22]. In contrast, *clpP*-recessive mutations were observed and identified as the cause of infertility and sensorineural deafness of Perrault syndrome [23,24].

**Figure 1:**
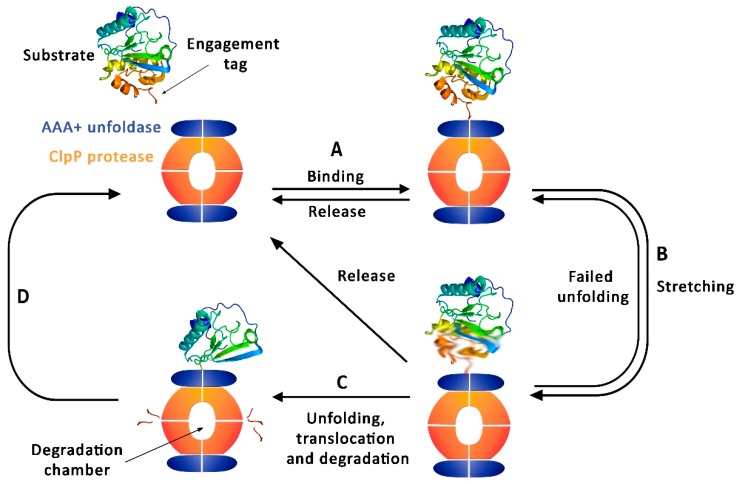
Cycle of ATPases associated with diverse cellular activities (AAA+) caseinolytic protease proteolytic subunit (ClpP) complex. (**A**) Substrate selective binding by the AAA+ unfoldase. (**B**) Sequence of ATP-driven stretching, leading to a strained structure or alternatively to a substrate release. (**C**) Successful unfolding leading to translocations and degradation. (**D**) Initiation of a new cycle.

As previously mentioned, presence of AAA+ unfoldases is required for the efficient action of this protease family. The Clp ATPases filter, unfold and introduce in the proteolytic chamber a range of proteins according to the situation following different regulatory processes (Figure 1) [25,26]. This target identification is often performed by inherent or added sequence motifs accessible either in the N or C terminus of these substrates [8].

The substrate selection will always rely on the specific unfoldase, and thus the common presence of diverse ATPases offers a tight regulation of the ClpP activity in cells [27]. Furthermore, the existence of adaptors binding to the Clp ATPases upon signals or stresses, influencing in this way the substrate choice, enlarges the control of this regulation. SspB, RssB and UmuD for *E. coli* caseinolytic protease subunit X (ClpX) [28,29,30], ClpS for *E. coli* caseinolytic protease subunit A (ClpA) or MecA for *B. subtilis* caseinolytic protease subunit C (ClpC) are some examples of these adaptors [31,32]. Recent identification of small anti-adaptor proteins, able to regulate the activity of the adaptors themselves, added an extra layer of complexity for control and fine-tuning of ClpP protease [33]. For further information on adaptor proteins regulating bacterial proteolysis, the excellent review by Battesti and Gottesman is recommended [34].

## 2. Structure of ClpP

**Figure 2:**
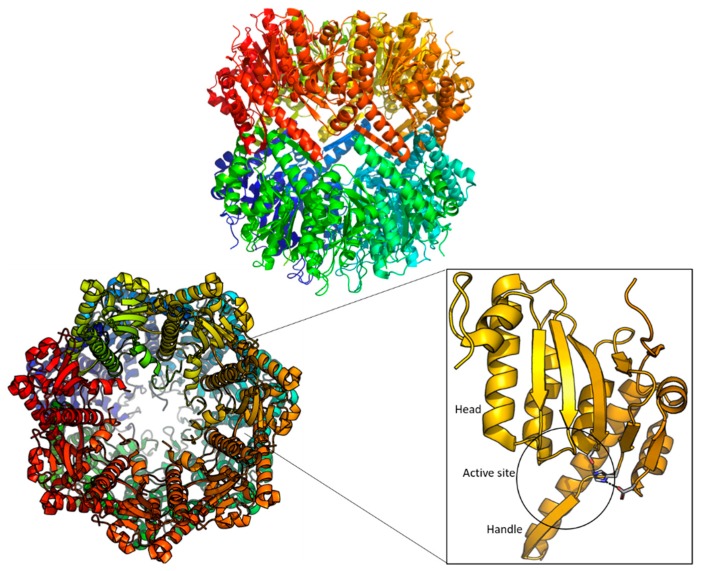
Structure of the full *E. coli* ClpP tetradecamer (top: side view, bottom left: top view) and the ClpP monomer (bottom right). Every monomer is shown in a different color in cartoon representation. The monomer is shown to emphasize the active site and the catalytic triad (S172, H122, D171) is shown in grey stick representation. (PDB ID: 1YG6).

The ClpP protease forms a tetradecameric cylinder with two heptameric rings longitudinally aligned (Figure 2). The axial pore of each ring (~10 Å diameter) acts as the entrance to the interior of the proteolytic chamber (~50 Å diameter) with the 14 active sites [35]. Each protomer, with a size ranging the 193-277 residues depending on the species, consists of a C terminus, an N terminal loop, a handle and a head domain (Figure 2). The handle domains of each heptameric ring are oriented to the same face, composing the interface that assembles the tetradecamer when the two handle faces bind to each other. When the tetradecamer is formed, the globular head domains comprise the main body of the ring, with most of their surface, including the active site, allocated in the interior of the barrel (Figure 2) [36]. Finally, the N-termini are highly flexible and are located in the axial region. Each of the active sites, contains the canonical Serine-Histidine-Aspartate catalytic triad [35].

ClpP tetradecamer in *E. coli* is formed by 14 identical protomers. Nevertheless, it can be found as a homomeric or heteromeric assembly in organisms were different isoforms of ClpP are present, as it is the case of ClpP1P2 in *Mycobacterium tuberculosis*, where each ring is formed with 7 protomers of a different paralog [37]. Other examples of heteromeric assembly include many cyanobacteria mixing ClpP and ClpR, an inactive variant of ClpP, leading to different types of tetradecameric complexes [38]. *Pseudomonas aeruginosa* also presents ClpP1 and ClpP2 isoforms, however in this case, they only form separate tetradecamers which perform distinct functions in the cell [39].

**Figure 3:**
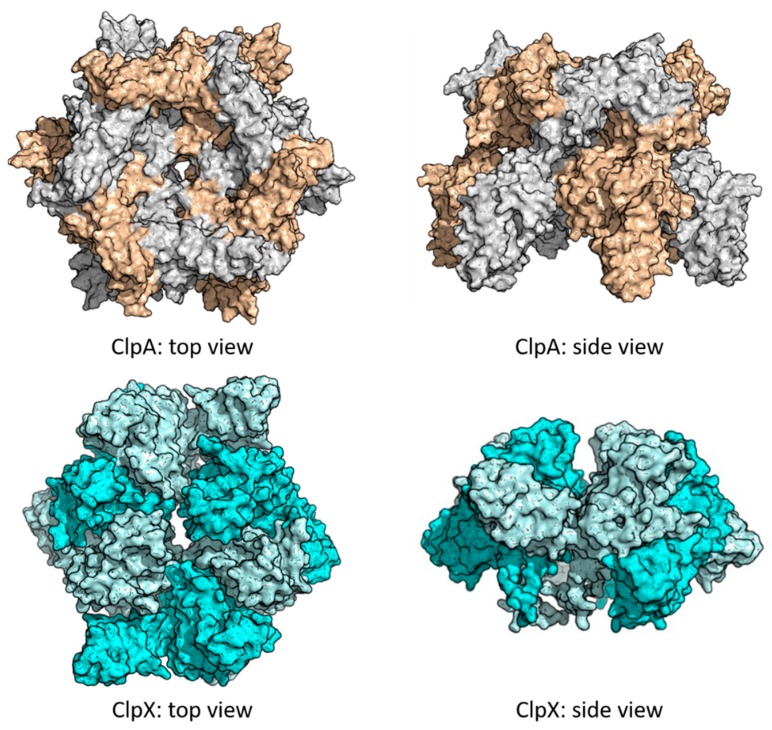
Top and side view of *E. coli* caseinolytic protease subunit A (ClpA) (top) and *E. coli* ClpX (bottom) hexamers. The ClpA hexamer is shown in grey and wheat surface representation. The structure was obtained through homology modeling, using the *B. subtilis* caseinolytic protease subunit C (ClpC) monomer as template (PDB ID: 3PXI) in SWISS-MODEL [42]. The subunits in the ClpC hexamer (PDB ID: 3PXG) where then replaced with the ClpA monomers, and the structure was then relaxed through energy minimization. The caseinolytic protease subunit X (ClpX) hexamer is shown in cyan and light cyan surface representation. Missing loops were modeled using Modeller 9.17 [43].

The peptidase activity of ClpP, characteristic of a chymotrypsin-like serine protease, results in peptides of 7-8 residues length, cutting after non-polar residues [27,40,41]. However, for efficient degradation of long peptides and proteins, as mentioned before, binding with an unfoldase partner such as ClpX or ClpA is needed. These chaperones belong to the HsP100 class of the AAA+ superfamily and act as a cap for the ClpP cylinder. Despite the existence of different Clp ATPases across different organisms, all share the structure of a hexameric complex with a central pore used as a channel to translocate the substrates into the proteolytic chamber (Figure 3).

They bind to seven grooves of about 10 Å of diameter, mainly composed of conserved hydrophobic residues and are compatible with the specific loop regions of the AAA+ chaperones as it is the case of IGF and IGL loop of ClpX and ClpA of *E. coli* respectively [44,45]. Thanks to these loop-groove interactions, the mismatch between the heptameric symmetry of the ClpP complex and the hexameric conformation of the chaperones is evaded. This mismatch has been suggested to be a result of evolution, which allows the chaperone to rotate around the ClpP complex, improving the efficiency of the unfolding and translocation [46]. It is also commonly accepted that the binding with the chaperone induces a conformational change in ClpP, turning a closed and inactive conformation into an open and proteolytically active conformer which accepts large peptide chains in the degradation chamber [45]. Further information about ClpP unfoldases structure and mechanistic can be found in a recent and comprehensive review by Olivares et al. [47].

With this information in mind, analogy with the well-known 20S peptidase could be easily done. ClpP and 20S peptidase share a self-compartmentalized nature, composed by aligned heptameric rings, with narrow axial pores leading to an inner proteolytic chamber with up to 14 catalytic sites. Need of association to ATP-unfoldase partners for an efficient proteolysis is also a common feature. However, they also display key differences. First, the size of proteasome is notably larger. Despite the residue count of its protomers and diameters of pores and inner chamber are comparable to the ones of ClpP, proteasome complex is formed by four heptameric rings, two of which have an exclusively structural nature. Furthermore, the active sites of 20S peptidase contain a catalytic threonine instead of the Ser-His-Asp of ClpP. For an in-depth comparison of ClpP and proteasome, readers are referred to the review published in by Liu et al. in 2014 [48].

## 3. ClpP as a Drug Target

### 3.1. ClpP in Bacteria and Parasites

ClpP started attracting attention due to its potential as an antibacterial target in the late 90s, when its direct relationship with bacterial virulence was proven for Gram-positive *S. aureus* and *Listeria monocytogenes* [49,50], together with Gram-negative *Salmonella Typhimurium* [51].

The ClpP of *S. aureus* is characterized most extensively. Direct relation between its virulence and ClpP function was demonstrated by Mei et al. when they performed a transposon-based mutagenesis screen, evaluating the virulence of the deletion strains in a murine model, identifying between others the ClpX ortholog [49]. Follow-up experiments enlarged the scope to ClpP itself, when *clpP*- and *clpX*-deficient mutants showed lack of virulence in a murine skin abscess model [10]. The same study established that the connection between ClpP function and the *S. aureus* virulence did not rely only on the regulation of stress responses, but also on the secretion of α-haemolysin (*hla* gene) and other effectors by the regulation of the accessory gene regulator (*agr*) locus [10]. Subsequent research related ClpP with the iron-regulated determinants (Isd) system regulation, which extracts heme-iron from the host hemoglobin and is fundamental for *S. aureus* pathogenesis [52].

In case of *L. monocytogenes*, Gaillot et al. established the essential ClpP role in the survival and growth of this bacteria under stress conditions [50]. Thus, the intracellular parasitism of *L. monocytogenes* is unfeasible, being susceptible to the bactericidal activity of the host [50]. From the two isoforms existing in this bacterial species, ClpP2 was found necessary for the Listeriolysin O factor expression [53]. The immunodominant virulence factor allows bacterial escape from the phagocytic vacuoles from the host and promotes intracellular growth [54].

Other Gram-positive pathogens where ClpP affects bacterial viability are *Streptococcus pneumoniae* and *Enterococcus faecalis*. In *S. pneumoniae* Δ*clpP* strains were unable to invade the lungs, lost the ability of colonizing the nasopharynx in mouse models and had lower survival rates in murine macrophages [55]. Furthermore, *clpP*-deficient mutant was found to be susceptible to nitric oxide and other oxidative stress [56]. In *E. faecalis*, deficiency of ClpP or Clp unfoldases led to loss of virulence in an invertebrate model [15].

The first Gram-negative ClpP studied was *S. Typhimurium*. Through a transposon-based mutational analysis, a Δ*clpP* strain unable to grow in a mouse model of typhoid fever was detected [51]. Subsequent studies explained how the survival of that strain is compromised in presence of peritoneal macrophages and therefore impedes virulence [57]. More recently, Knudsen et al. found, using a transcriptomic analysis, how ClpP regulation of RpoS (RNA polymerase, sigma S) and CsrA (Carbon storage regulator A) factors indirectly affects *S. Typhimurium* virulence [58].

More recent examples of Gram-negative bacteria where ClpP plays a central role in pathogenesis are *P. aeruginosa* and *Legionella pneumophila* [13,16]. For *P. aeruginosa*, ClpX and ClpP2 were identified as part of the proteolytic network of the exopolysaccharide alginate biosynthesis, a characteristic marker for the onset of chronic lung infection in cystic fibrosis [13]. Furthermore, Bishop et al. established the relevant role of ClpP in the Cell Surface Signaling (CSS) pathway, which enables iron uptake during *P. aeruginosa* infection [59]. Likewise *L. monocytogenes*, *clpP*-deficient mutants in *L. pneumophila* showed impaired virulence and inability to escape endosome-lysosome pathway in mammalian cells [16].

In *E. coli*, where ClpP is responsible for the cleavage of proteins involved in metabolism, transcription factors, as well as in oxidative stress response and starvation; [10] Robinson et al. showed significant differences between growth curves of wild type and Δ*clpP E. coli* under nitric oxide-stress [60].

Opposed to the described bacteria, where ClpP influences the growth in stress condition and virulence but not the viability of the species, *M. tuberculosis* ClpP1P2 was found to be an essential target [14,61]. As mentioned before, ClpP1 and ClpP2 isoforms ensemble a tetradecameric complex with one heptameric ring of each analogue [37]. These isoforms together with the chaperones ClpX and ClpC1 were identified as essential for the pathogen viability and infectivity *in vitro* [14].

ClpP has not only been studied in bacteria but also in protozoal parasites as it is *Plasmodium falciparum*. This unicellular eukaryotic parasite is the causative agent of malaria in humans. *P. falciparum* adopts several cellular morphologies and different reproductive stages when it moves from the mosquito carrier to human hosts. This parasite ClpP is localized in the apicoplast, a plastid bound by four membranes located near to a mitochondrion. This organelle is needed for isoprenoids, fatty acids, heme and iron-sulfur clusters biosynthesis [62]. An analysis of the ClpP expression through the *P. falciparum* life cycle demonstrated overexpression in the stages in which *P. falciparum* colony increases and infects red blood cells (late trophozoite an early schizont) [63]. Accordingly, growth decrease by inhibition of ClpP by a β-lactone was observed [63], interfering with the progression from early to late schizont stage.

In summary, the relevance of ClpP protease in bacterial pathogenesis offers an untapped target for new antivirulence and antimicrobial drugs [17].

### 3.2. ClpP in Human Mitochondria

Even though eukaryotic ClpPs have been less studied, human ClpP plays a key role in the quality control system of mitochondria. Human ClpP (hClpP) shares a high degree of sequence identity with its bacterial orthologs and the obtained X-rays suggest a close resemblance to their structure [48,64]. While human ClpP active form is the common tetradecameric complex with ATP unfoldases as human ClpX (hClpX) associated, ClpP exists as an inactive single heptameric ring, that only fuses into the tetradecamer by induction of hClpX binding [65]. Similarly to bacterial ClpP, hClpP degrades misfolded, mistranslated or aggregated proteins to keep mitochondrial respiratory efficiency, as hClpP target proteins are involved in a number of processes like fatty acid, amino acid and energy metabolism [19,20,21,66]. Given the relevant role of hClpP, it is not surprising to find human conditions in which this protease is involved.

In 2013, Gispert et al. reported that *CLPP* null mice showed infertility, reduced reproductive organs, severe growth retardation, diminished spontaneous motor activity and a strong decrease in survival [24]. Interestingly, these phenotypes correspond to Perrault syndrome symptoms in humans. This is a genetic disorder where also patients suffer sensorineural hearing loss and, in case of females, ovarian dysfunction leading to diminished fertility while male patients do not display sterility issues. Developmental delay or intellectual disability are some of the neurological defects present in some patients with this condition. During the same year, by a combination of homozygosity mapping, linkage analysis and exome sequencing in three families, *CLPP* recessive mutations were identified in a Pakistani family with Perrault syndrome. In this way, mutation in *CLPP* joined the already described mutations in *HARS2*, *HSC17B4*, *LARS1* and *C10orf2* [67]. Along the subsequent years new cases appeared [68,69]. It is suggested that hClpP structural integrity is compromised by these mutations, leading to lower ClpP levels in mitochondria, affecting mitochondrial respiratory function and leading to the symptoms of the disease.

Two years later, Cole et al. correlated hClpP overexpression with development of acute myeloid leukemia (AML) [21]. Shortly after, its complex with hClpX was identified as a key player of the bioenergetics stress control and found crucial for metastasis broadening the scope for a number of carcinomas [22]. Rapid and unregulated proliferation is a characteristic of cancer cells, it demands a significant alteration of the energy metabolism, increasing reactive oxygen species (ROS) and inducing oxidative stress [70]. This stress is balanced by upregulation of a number of mitochondrial proteases, such as Lon or ClpP, elevating its respiratory function to handle the oxidative damage to different biomolecules and other impairments in the cell [71]. Therefore, overexpression of ClpP and other mitochondrial proteolytic machines, such as heat shock protein-90 (Hsp90), in various types of cancer is consistent [22,72].

However, *CLPP* knockdown studies have revealed a variety of results across the different cell types. In experiments on AML cell lines, cell growth and viability were drastically reduced [21]. Prostate adenocarcinoma cell lines showed cell cycle arrest together with inability for colony formation [22]. However, barely any inhibition of cell proliferation was observed for the non-metastatic breast adenocarcinoma [22]. Potential co-administration of ClpP modulators with chemotherapy is also suggested by the correlation between ClpP expression and cisplatin resistance recently reported by Zhang et al. [73].

## 4. ClpP Modulation

Due to the complexity of these AAA+ proteolytic machines, many pathways to alter the normal function of the ClpP complex have been explored. While orthosteric inhibitors that bind and block the catalytic triad of the ClpP monomers seems like the most obvious choice, several alternatives impeding interaction of the tetradecamer with the ATP-unfoldases or upregulation of these chaperones have been investigated.

### 4.1. Targeting ClpP-ATPases Interaction

**Figure 4:**
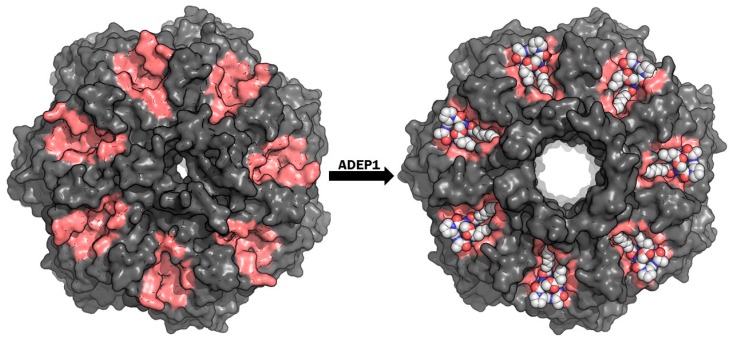
Top view of *E. coli* ClpP in unbound state (**left**) (PDB ID: 1YG6) and bound with acyldepsipeptide (ADEP)1 (**right**) (PDB ID: 3MT6). When bound, an increase in pore size can be observed which defines an active, non-selective conformation. The protein is shown in grey surface representation, with the ADEP1 binding pockets shown in pink. ADEP1 molecules are represented by white spheres.

Interestingly, the most advanced drug-based studies for ClpP modulation do not proceed from a medicinal chemistry small molecule research focused on enzymatic inhibition, but from a factor isolated in the fermentation broth of *Streptomyces hawaiiensis*, which is able to dysregulate ClpP function [74]. The original group of eight factors was discovered by Eli Lilly and Company, who reported a notable in vitro antibacterial activity against *Staphylococcus* and *Streptococcus* strains [75]. Later, Osada et al. reported a depsinopeptide with similar antibacterial activity in *E. faecalis* and *S. aureus* called enopeptin A [76]. However, no suggested mechanism of action or activity in vivo was described until 2005, when Brötz-Oesterhelt et al. characterized the original cyclic acyldepsipeptide (ADEP1) structures from Eli Lilly and identified ClpP as their target [74]. ADEPs do not inhibit ClpP proteolytic activity nor interact with the catalytic triads, instead, these cyclopeptides compete with and displace the ATP-unfoldase ClpX specific loop regions (IGF and IGL) that bind into the ClpP grooves on the apical regions. By interaction with those grooves or pockets allocated between the adjacent monomers, ClpP tetradecameric complex adopts the active conformation of the ClpXP complex, broadening the diameter of the axial pores while lacking the selective and regulatory function of ClpX (Figure 4) [77,78]. In this way, ClpP performs uncontrolled proteolysis leading to bacterial death [74]. A comprehensive review about ClpP dysregulation by ADEPs was recently published by Malik et al. [79].

**Figure 5:**
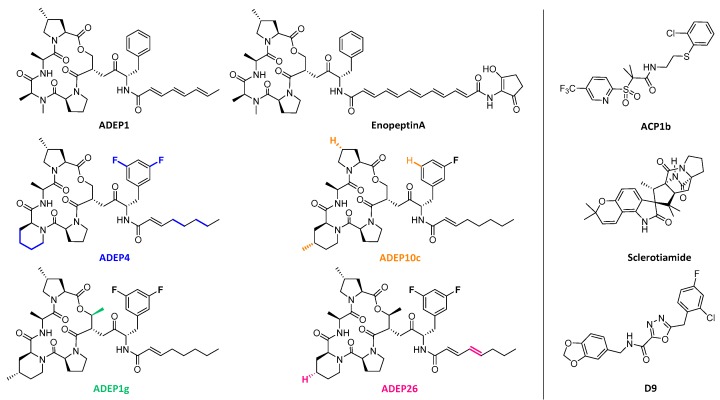
Chemical structures of ClpP activators. On the left, chemical structures of the mentioned ADEPs are shown. In the color matching their name, consecutive modifications respect to the previous ADEP are highlighted: the original ADEP1 and EnopeptinA (black), ADEP4 (blue), ADEP10c (orange), ADEP1g (green) and ADEP26 (pink). On the right side, alternative cores extracted from screening including ACP1b, Sclerotiamide and D9.

Improvement from the first ADEPs (ADEP1 and EnopeptinA, Figure 5), with poor ADME properties and antibacterial activity limited to Gram-positive pathogens [74,76], was progressively achieved by medicinal chemistry investigations. First, the inclusion of a pipecolate moiety to increase the rigidity of the depsipeptidic cycle, the replacement of a phenylalanine by its 3,5-difluorinated analogue and the partial saturation of the side chain led to a 160-fold increase of antibacterial activity and a boost of bioavailability and chemical stability (ADEP4, Figure 5) [74,80]. This compound was tested in mouse models proving its efficacy against *S. aureus*, *E. faecalis* and *S. pneumoniae*, proving as well desirable ADME properties with moderate to high clearance and distribution, with a half-life of 1–2 h in mice and dogs [74]. In contrast to other bacteria where these compounds activate an uncontrolled proteolysis leading to cell death, ADEP4 was able to eradicate *M. tuberculosis* by only preventing binding between ClpX and essential ClpP1P2 [81]. Furthermore, co-administration of ADEP4 and rifampicin in animal models eradicated *S. aureus* chronic biofilm infections [82]. Then, further constrains in the cycle were added by methylation of the piperidine and the cycled serine function were translated in improved antibacterial activity (ADEP10c and ADEP1g, Figure 5) [83,84]. Despite the success of the described compounds against Gram-positive bacteria, no ADEP with activity against Gram-negative was discovered until 2016 [85]. Goodreid et al. reported a set of compounds (ADEP26, Figure 5) which is not only able to kill *S. aureus*, *E. faecalis*, *S. pneumoniae*, *B. subtilis* and *Listeria innocua*; but also displayed antibacterial activity against Gram-negative *Neisseria meningitidis* and *Neisseria gonorrhoeae* [85]. With the aim of identifying new scaffolds with a similar mode of action to the ADEP family, a high-throughput screening (HTS) with *E. coli* ClpP was run [86]. Five clusters were identified, after lead optimization ACP1b (Figure 5) was the most developed structure, with a remarkable bactericidal activity against *S. pneumoniae*, *S. aureus* and Gram-negative *N. gonorrhoeae*, *N. meningitidis*, *P. aeruginosa*, *L. monocytogenes* and *Haemophilus influenza* [86]. Another scaffold of these activators was found by Lavey et al. in a fluorescence-based assay again with *E. coli* ClpP [87]. Despite sclerotiamide does not possess a comparable potency to its counterparts, the unique bicycle-[2.2.2]-diazoctane core opens a new and interesting spectrum for functionalization [87]. Finally, Stahl et al. discovered a species-selective activator for human ClpP (D9, Figure 5) [88]. This small molecule contains a halogenated benzyl moiety that binds to a unique aromatic amino acid network in the hClpP groove [88].

While ADEPs and the other new activators bind to the ClpP axial pockets of the tetradecamer, activating an uncontrolled proteolysis and impeding the connection with the AAA+ unfoldase, other families of compounds are known to target the unfoldases themselves.

**Figure 6:**
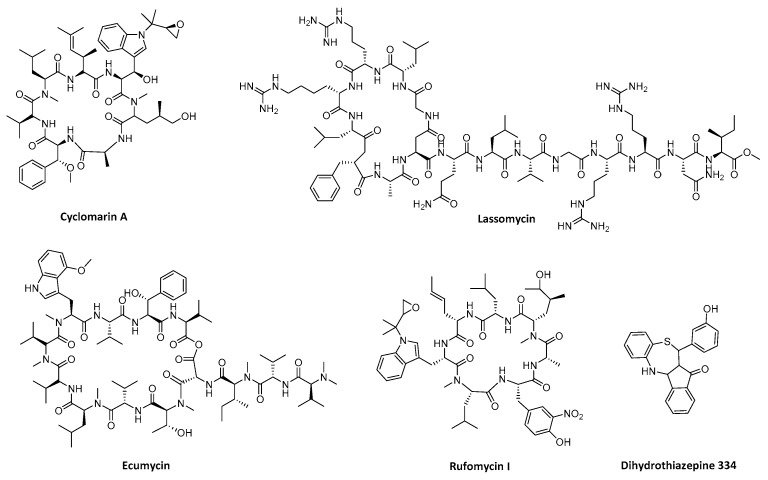
Chemical structures of compounds targeting ATP-unfoldases.

This strategy has been broadly investigated in *M. tuberculosis* due to the essentiality of ClpP1P2 and its unfoldase ClpC1 in this pathogen. Large cyclic peptides such as cyclomarin A [89], lassomycin [90]. ecumicin and rufomycin (Figure 6) have shown their antibacterial action even with multidrug resistant *Mtb* strains [91,92]. Although their exact mode of action is not yet unraveled, they all target ClpC1, uncoupling its action from the ClpP1P2 proteolytic activity. Another recent example of ATP-unfoldase targeting is the dihydrothiazepine 334 (Figure 6) described by Fetzer et al. [93]. This compound was able to interact with *S. aureus* ClpX and provoke its deoligomerization. Toxin production levels of multidrug-resistant *S. aureus* were reduced and virulence attenuated.

### 4.2. ClpP Inhibition

**Figure 7:**
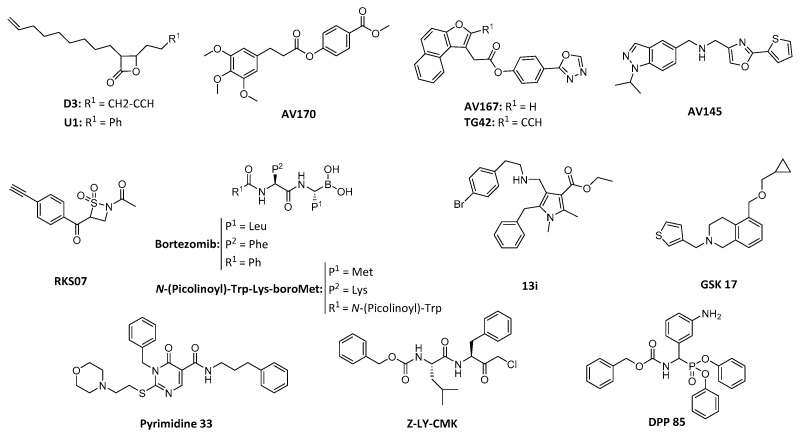
Chemical structure of reported ClpP inhibitors.

A classical approach of designing a drug able to inhibit the catalytic triad of the serine protease has also been explored with uneven success. In 2008, a group of β-lactones were identified to inhibit *S. aureus* ClpP when Böttcher and Sieber used activity-based protein profiling to identify the targets involved in their antivirulence action [94]. From them, D3 (Figure 7) stood out as the most potent and selective irreversible inhibitor [95]. Optimization of this family led to synthesis of U1 (Figure 7), improving D3 potency between a 3 and a 5-fold [96]. Further characterization showed the ability of these compounds to also inhibit ClpP and therefore reducing the expression of extracellular virulence factors not only in *S. aureus* but also in *L. monocytogenes* [97]. β-Lactones have also displayed ClpP inhibition in other pathogens such as *P. falciparum* or *M. tuberculosis* [63,98]. Despite successful results in in vitro assays, the reduced plasma stability due to hydrolysis of the cyclic ester and their generally poor selectivity has limited their clinical development [99].

Thus, further investigations in search of a new class of ClpP inhibitors was carried out by Sieber et al., who conducted a HTS with more than 137.000 compounds [99]. The main result of this study was the identification of a phenyl ester AV170 (Figure 7), which triggered deoligomerization of the ClpP tetradecamer by covalent binding to the catalytic serine [99]. AV167 (Figure 7) also drew attention due to its inhibition of the hClpP [99]. A recent follow up study evolved this compound into a selective inhibitor of the human ClpP by addition of an alkyne in the naphtofurane moiety (TG42, Figure 7) [100]. However, their improved potency, pharmacokinetics and plasma stability were counteracted by their lower anti-virulence activity. In addition, no attempt of structure development was able to maintain the inhibitory activity [99]. A reinvestigation of the same HTS campaign with less strict selection criteria gave as a hit the first non-covalent binding inhibitor of *S. aureus* ClpP [101]. This compound AV145 (Figure 7) does not possess a substrate-like chemical structure and consequently does not bind to the active site but to the handle region of each monomer. This binding induces a novel and inactive conformation of the tetradecameric complex [101].

**Figure 8:**
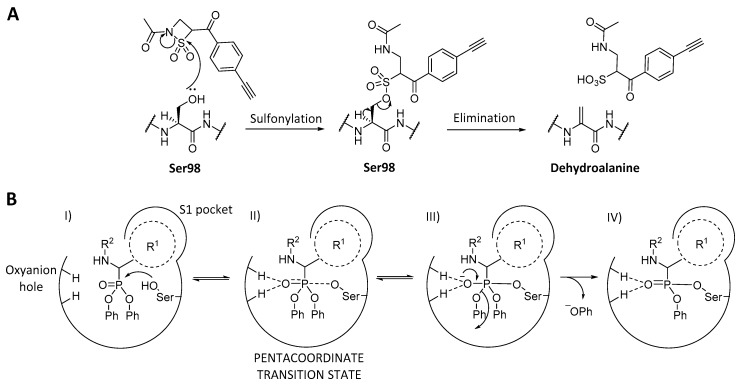
(**A**) Mechanism of action of RKS07 [102], a β-sultam undergoing sulfonylation and subsequent elimination to transform the catalytic serine into an inactive dehydroalanine. (**B**) Inhibitory mechanism of a diphenyl phosphonate inhibitor with a serine protease. (I) The unreacted inhibitor enters the active site, with the R^1^ moiety filling the S1 (recognition) pocket while the phosphonate stays at a reachable distance from the catalytic serine and the oxyanion hole. (II) Nucleophilic attack of the serine to the phosphonate induced by the interaction with the oxyanion hole residues, forming the pentacoordinate transition state. (III) Release of the phenolate group due to the recovery of tetrahedral geometry. (IV) Final configuration leading to irreversible inhibition of the serine protease.

A new inactivation mechanism was discovered by Gersch et al. when they found β-sultams (RKS07, Figure 7) to selectively modify ClpP catalytic serine [102]. Nucleophilic attack of Ser98 to the cyclic sulfone, followed by elimination of the sulfonylated serine results in formation of dehydroalanine (Figure 8A). The alteration of the catalytic residue triggers disassembly of the active complex into two inactive heptameric rings [102].

Strong evidence of the potential of boron-derived compounds as ClpP inhibitors has been shown in *M. tuberculosis*. Bortezomib (Figure 7), a known 26S proteasome inhibitor studied in treatment for cancer, demonstrated to inhibit ClpP1P2 [103]. However, its high cost, poor pharmacokinetics, short half-life and proteasome inhibition limited the use of this drug at *M. tuberculosis* treatment [103]. Substrate-based boronates (*N*-(picolinoyl)-Trp-Lys-boroMet, Figure 7) by Akopian et al. also displayed selective targeting of ClpP1P2 inducing growth inhibition. New pyrrole cores (13i, Figure 7) were recently identified as ClpP1P2 inhibitors by Liu et al. after an in silico driven exploration, showing antibacterial properties and room for optimization [104]. Furthermore, a HTS setting for detection of *Mtb* ClpC1P1P2 complex inhibitors identified two hits [105]. Despite not showing antibacterial properties and having unclear mechanisms of action, both inhibitors showed a new chemical structure for ClpP inhibitors (GSK 17, Figure 7) and forthcoming use of broader libraries envisages a promising future for detection of new inhibitors for this pathogen [105]. Inhibition of *P. falciparum* ClpP has also been achieved with a novel class of ClpP inhibitors [106]. A family of pyrimidines (33, Figure 7) showed inhibition of growth and segregation of the apicoplast leading to parasite death [106].

Finally, although ClpPs of several organisms have been targeted, inhibition of Gram-negative bacteria ClpP remains barely untapped. The co-crystallized chloromethyl ketone (Z-LY-CMK, Figure 7) in *E. coli* ClpP was the only reported inhibitor for this pathogens until not long ago [107]. Recently, based on that co-crystallized structure, a new class of ClpP inhibitors with an α-amino diarylphosphonate warhead was reported by our group [108]. This family of compounds is a classic example of irreversible inhibitors of serine proteases [109,110,111,112,113,114]. Their mechanism of action consists of a nucleophilic attack of the catalytic serine to the phosphonate, which undergoes release of a phenolate group to recover the tetrahedral geometry from the pentacoordinate transition state (Figure 8B).

Nevertheless, from a screening of a focused library of about 150 compounds, 14 identified inhibitors displayed an unexpected reversible binding. This suggests that the phosphonate does not reach an accessible distance for the Ser98 to undergo nucleophilic attack, but still allows reversible blockage of the active site [108]. One of the reported hits (DPP 85, Figure 7), was also able to delay and decrease growth under nitric oxide-stress. This reinforced the results obtained by Robinson et al. [60], where the Δ*clpP* mutant strain displayed impaired growth under the nitric oxide conditions. Furthermore, administration of DPP 85 to the Δ*clpP* strain did not produce an additional effect, consistent with a ClpP mediated effect. We hope this study encourages further investigation in the ClpP of Gram-negative bacteria.

## 5. Remarks and Future Challenges

The bacterial ClpP protease is a promising novel drug target, not included in the large antibacterial screenings of pharmaceutical companies in the 1990s and 2000s because it is not essential in most of pathogens. The same lack of essentiality, poses ClpP as an adequate antivirulence target, as it has potential to disarm pathogens without killing them, which could avoid triggering of resistance development. In this way, modulation of ClpP joins a group of emerging antivirulence pathways such as disruption of quorum sensing and hindrance of biofilm formation [115,116,117].

Despite the amount of studies providing information about the mechanism and structure of ClpP, ClpX or ClpA, no high resolution structures of their complex, in the process of unfolding or translocating a protein, are available yet. In the same way, while the role of ClpP in pathogens has been broadly studied, understanding of hClpP influence in human mitochondria remains limited. Human ClpP connection with cancer and Perrault syndrome will be the focus of many studies in the coming years.

Although the number of reported molecules targeting the modulation of ClpP is considerable, no compound has reached the clinic yet, indicating the early stage of the research in this protease. Interestingly, from all the compounds modulating bacterial ClpP function, almost no studies include a description of their action in human ClpP. Given the high sequence conservation of ClpP and with the ultimate goal of developing an antimicrobial drug, comprehensive selectivity studies of ClpPs in different species should be considered.

Currently, the most successful and advanced strategy has been to target the protein-protein interaction between ClpP and its ATPases. Allosteric activation of ClpP, triggering uncontrolled proteolysis, presents an exceptional opportunity for drug development.

Finally, the majority of efforts in the ClpP research were focused on the Gram-positive bacteria and mycobacteria. Desperate need of new therapeutic options for multidrug resistant bacteria and the abundance of Gram-negative among the list of priority targets described by the World Health Organization: ESKAPE (acronym for Gram-positive *E. faecalis* and *S. aureus* and Gram-negative *Klebsiella pneumoniae, Acinetobacter baumannii, P. aeruginosa* and *Enterobacter*) point at the urgency of this matter [118]. Even though Gram-negative bacteria double membrane and resistance mechanisms pose the serious challenge, additional efforts in the Gram-negative ClpPs is encouraged.

## Abbreviations

AAA+	ATPases associated with diverse cellular activities
ADEP	Acyldepsipeptide
*agr*	Accessory gene regulator
AML	Acute myeloid leukemia
*B. subtilis*	*Bacillus subtilis*
ClpA	Caseinolytic protease subunit A
ClpC	Caseinolytic protease subunit C
ClpP	Caseinolytic protease proteolytic subunit
ClpS	Caseinolytic protease subunit S
ClpX	Caseinolytic protease subunit X
CSS	Cell surface signaling
*E. coli*	*Escherichia coli*
*E. faecalis*	*Enterococcus faecalis*
hClpP	Human ClpP
hClpX	Human ClpX
HTS	High-throughput screening
Hsp90	Heat shock protein-90
Isd	Iron-regulated determinants
*L. monocytogenes*	*Listeria monocytogenes*
*L. pneumophila*	*Legionella pneumophila*
*M. tuberculosis*	*Mycobacterium tuberculosis*
*Mtb*	*Mycobacterium tuberculosis*
Mpa	*Mycobacterium* proteasome-associated ATPase
*N. gonorrhoeae*	*Neisseria gonorrhoeae*
*N. meningitidis*	*Neisseria meningitidis*
*P. aeruginosa*	*Pseudomonas aeruginosa*
*P. falciparum*	*Plasmodium falciparum*
Pan	Proteasome-activating nucleotidase
ROS	Reactive oxygen species
*S. aureus*	*Staphylococcus aureus*
*S. pneumoniae*	*Streptococcus pneumoniae*
*S. Typhimurium*	*Salmonella Typhimurium*
SDS-PAGE	Sodium dodecyl sulfate polyacrylamide gel electrophoresis
WT	Wild-type
Z-LY-CMK	Benzyl ((*S*)-1-(((*S*)-4-chloro-1-(4-hydroxyphenyl)-3-oxobutan-2-yl)amino)-4-methyl-1-oxopentan-2-yl)carbamate
Δ*clpP*	*clpP*-defective mutant
